# Factors Influencing Alzheimer’s Disease Risk: Whether and How They are Related to the APOE Genotype

**DOI:** 10.1007/s12264-021-00814-5

**Published:** 2022-02-11

**Authors:** Rong Zhang, Xiaojiao Xu, Hang Yu, Xiaolan Xu, Manli Wang, Weidong Le

**Affiliations:** Institute of Neurology, Sichuan Academy of Medical Sciences, Sichuan Provincial People’s Hospital, University of Electronic Science and Technology of China, Chengdu, 611731 China

**Keywords:** Apolipoprotein E, Alzheimer’s disease, Ethnicity, Diet, Geographic factor, Aging, Gender

## Abstract

Alzheimer's disease (AD) is the most prevalent neurodegenerative disease featuring progressive cognitive impairment. Although the etiology of late-onset AD remains unclear, the close association of AD with apolipoprotein E (APOE), a gene that mainly regulates lipid metabolism, has been firmly established and may shed light on the exploration of AD pathogenesis and therapy. However, various confounding factors interfere with the *APOE*-related AD risk, raising questions about our comprehension of the clinical findings concerning *APOE*. In this review, we summarize the most debated factors interacting with the *APOE* genotype and AD pathogenesis, depict the extent to which these factors relate to *APOE*-dependent AD risk, and discuss the possible underlying mechanisms.

## Background

Alzheimer's disease (AD) is one of the most prevalent and influential neurodegenerative diseases, characterized by typical pathological findings of beta-amyloid (Aβ) and tau plaques [1, 2]. Featuring irreversible and progressive deterioration of cognitive function and mainly affecting the elderly, AD imposes an enormous burden on patients, communities, and healthcare systems. Unfortunately, as life expectancy increases, the population of AD patients is expanding rapidly. The number of AD patients in the USA is estimated to grow from 4.7 million to 13.8 million from 2010 to 2050 [3]. Other countries are believed to be confronted with a similar impact of AD.

**Fig. 1:**
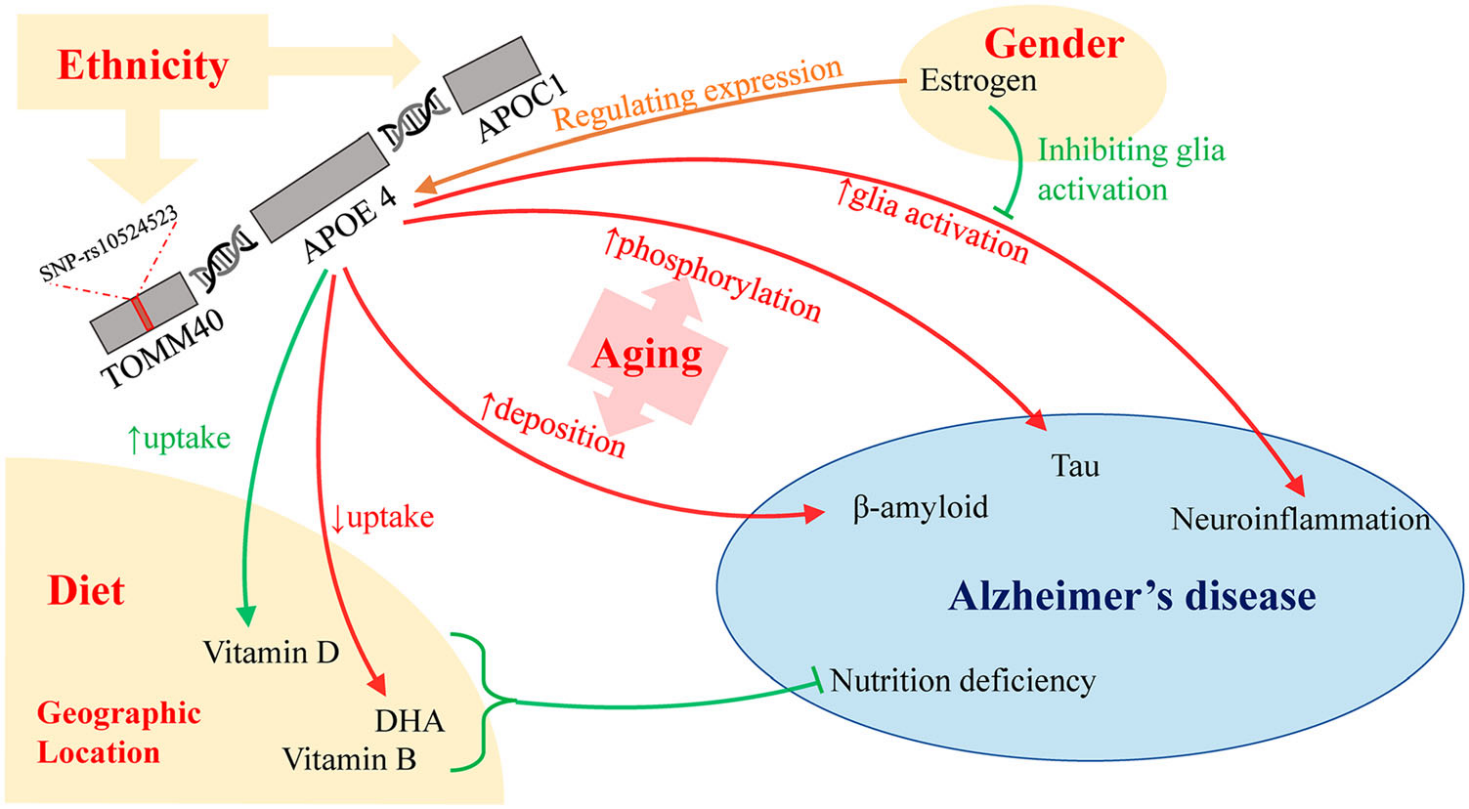
Interaction of confounding factors with the APOE ε4 allele and Alzheimer’s disease (AD). APOE ε4 influences AD through regulation of β-amyloid and tau deposition, neuroinflammation, and neuronal nutrition. Confounding factors may influence the ε4-AD association though the following mechanisms: ethnicity impacts the local ancestry of the APOE gene and thus AD risk; gender acts with the effects of hormones, mainly estrogen, to change APOE expression and neuroinflammation; aging directly enhances proteinopathy; diet and geographic location alter the nutritional status, jointly with APOE. APOE, apolipoprotein E; TOMM40, Translocase of the Outer Mitochondrial Membrane 40; APOC1, apolipoprotein C1; DHA, docosahexaenoic acid.

Apolipoprotein E (APOE) is a multifunction protein that plays a crucial role in the intercellular and interstitial transport of lipid and the mediation of dynamic lipid levels and lipid metabolism [4]. APOE fulfills its function by forming a close connection with lipoproteins and their receptors. Besides, APOE is also involved in neurophysiological processes such as synapse development and remodeling. Two vital single nucleotide polymorphisms (SNPs) located in *APOE* coding regions, rs429358 (C>T) and rs7412 (C>T), define the three major subtypes of *APOE* allele, ε2, ε3, and ε4. Alteration of ε2, ε3, and ε4 confers crucial variation on the protein structure of APOE, its physiological function, and its effect on related diseases [5, 6]. Besides its crucial effect on lipid metabolism, ε4 is the first, by far the most relevant and the most intensively studied risk gene for late-onset AD [6, 7]. *APOE* ε4 carriers have a higher lifetime incidence of AD and an earlier onset. In rough estimation, Farrer* et al.* found that individuals who carry one ε4 allele bear a 2–4 fold AD risk, and those with two copies of ε4 have an 8–12 fold AD risk [8].

Since the close association of *APOE* and AD was explicitly established in 1993, many studies have explored the underlying mechanism. Although the exact mechanism remains obscure, it is widely acknowledged that APOE is extensively involved in various pathologic processes of AD. (1) Aβ-dependent pathways: *APOE*-related AD risk can be largely attributed to an alteration of protein deposition, for the *APOE* genotype is no longer significantly associated with the clinical phenotype after controlling for AD pathology [9]. *APOE* ε4 carriers and ε4 knock-in mice both manifest exacerbated Aβ proteinopathy [10, 11]. Experiments showed that Apoe/APOE co-precipitates with Aβ in mice and AD patients [12], APOE ε4 may facilitate the aggregation both *in vitro* and *in vivo* [13, 14]. Researchers also reported the more rapid formation of Aβ oligomer as well as slower clearance of Aβ in ε4 knock-in rats, which corresponds with the results from studies in *APOE* ε4 cells [15–17]. (2) Aβ-independent pathways: APOE ε4 can up-regulate neuroinflammation, as reported in ε4 carriers and knock-in mice [18, 19], possibly through regulation of glia [20–22]. APOE ε4 is harmful to the integrity of the blood-brain barrier in mice [23], and blood-brain barrier impairment of the hippocampus and medial temporal lobe, correlated with cognitive dysfunction, has been reported in human ε4 carriers. Besides, the *APOE* genotype may alter the production of brain-derived neurotrophic factor and neuroprotective sirtuin, as well as energy expenditure [24–26].

*APOE*-related AD risk can be influenced by various factors, such as ancestry, gender, environment, and diet. These factors may exert their influence through distinct mechanisms such as regulation of transcription or expression of *APOE*, alteration of lipid metabolism, impacting the AD pathological process in which *APOE* is deeply involved. Previous epidemiological research provides abundant evidence to understand how these factors interact with *APOE*-related AD risk and the possible physiological explanation of these interactions. Here, we provide an overall review of the most debated confounding factors and discuss how they are connected to *APOE* and the pathogenesis of AD (Fig. 1).

## Ethnicity and APOE

The rough frequency ranges of the *APOE* ε2/3/4 alleles are (0–7)%/(69–85)%/(4–40)% across the world [7], but this ratio varies significantly among different ethnicities (Table 1). Generally, the frequency of the ε2 allele is relatively constant, which leads to the negative correlation of the ε3 and ε4 alleles [27]. The *APOE* ε4 allele comprises a larger proportion in Central Africa (40%), Oceania (37%), and Australia (26%), while in Europe and Asia, the ε4 allele frequency ranges from 10% to 25%, roughly positively related to the latitude of residence [7].

**Table 1:** Frequency of APOE ε4 allele in different ethnic groups.

Ethnicity	Country	APOE ε4 frequency in the entire population	AD risk OR (ε3ε4)	AD risk OR (ε4ε4)	References
African	USA	19.0	1.1	5.7	Farrer L.A. *et al*., 1997 [8]
Caucasian	Multinational	13.7	2.7	12.5	Farrer L.A. *et al.*, 1997 [8]
Hispanic	Multinational	11.0	2.2	2.2	Farrer L.A. *et al.*, 1997 [8]
Japanese	Japan	8.9	5.6	33.1	Farrer L.A. *et al.*, 1997 [8]
Chinese (Han)	China	13.5	2.7	8.3	Tan L. *et al*., 2013 [28]
Indian	India	7.0–12.7	4.2	4.8	Agarwal R. *et al.*, 2014 [29]
Chilean	Chile	19	2.4	12.8	Quiroga P. *et al*., 1999 [30]
Iranian	Iran	2.6–6.7	3.7	7.5	Abyadeh M. *et al*., 2019 [31]

APOE, apolipoprotein E; AD, Alzheimer’s disease; OR, odds ratio.

The ethnic background has a certain impact on AD risk, based on numerous epidemiological studies (Table 1). A 7-year longitudinal study based on the multi-ethnic population in New York established that, compared with Caucasians, African-American people [hazard ratio (HR) = 2.6] and Caribbean Hispanic people (HR = 2.3) are confronted with a significantly higher risk of AD [32]. Later, the cohort study conducted in Northern California included six ethnic groups further demonstrated that Asian Americans have the lowest incidence of AD, followed by Caucasians, Pacific Islanders, and Latin Americans (HR = 1.25–1.29), then American Indians (HR* =* 1.43), and the most affected African Americans (HR = 1.73) [33]. Kevin and his team summarized 28,027,071 beneficiaries of the Medicare Fee-for-Service to estimate the prevalence of AD and related dementias in different subgroups. The result showed that the order of ethnicities with prevalence from low to high is Asian and Pacific Islanders (8.4%), American Indians and Alaska Natives (9.1%), non-Hispanic Caucasians (10.3%), Hispanics (12.2%), and African Americans (13.8%) [34]. Since these studies included and analyzed several confounding factors such as educational level, vascular diseases, and other comorbidities, the authors made it clear that the inequalities in AD incidence most likely result from the diversity of ethnic genetic backgrounds.

The meta-analysis by Farrer and colleagues found that African and Hispanic ε4 carriers, compared with Caucasian ε4 carriers, have a lower *APOE*-related AD risk [8]. Meanwhile, Japanese ε4 carriers have an even higher odds ratio than Caucasian Americans. The data roughly showed that, with respect to ancestry difference, *APOE* ε4 frequency is inversely associated with the ε4-related AD risk, implying that the *APOE* gene polymorphism partially contributes to the vulnerability to diseases like AD. In two cohort studies by Tang *et al.* and Evans *et al.*, *APOE* ε4 was found to cause a lower increase in AD incidence in Africans than in Caucasians, despite African Americans bearing higher basal AD incidence [32, 35].

The mechanisms underlying the distinct ancestry-specific ε4-related AD risk remains unclear, but genetic research has provided insightful explanations regarding this issue. By comparing the association between SNPs and odds ratios in respect of AD, researchers have found that variations in the region surrounding the *APOE* gene accounts for most of the ethnicity-specific *APOE* effect on AD. Blue *et al.* compared 3,067 Caribbean Hispanics with 3,028 Europeans concerning the *APOE* genotype, local ancestry, genome-wide ancestry, and AD risk [36]. They discovered that local ancestry shows the strongest association (odds ratio, OR* =* 0.61) with AD risk other than the ε4ε4 genotype (OR* =* 8.59), while the impact of genome-wide ancestry is much less (OR* =* 1.004). Rajabli *et al.* used 5,496 African American and 389 Puerto Rican individuals to analyze the effect of local ancestry and global ancestry on *APOE* ε4-related AD risk. They found that only local ancestry has a significant influence (*P =* 0.019) [37]. Cornejo-Olivas *et al.* conducted genome-wide genotyping in the Peruvian population. They reported that the ancestry local to the *APOE* gene, rather than the whole genome background, contributes to the ε4-related AD risk [38]. Conversely, a few studies have reported that the heterogeneous ancestry-specific *APOE* ε4 effect may be derived from different genetic backgrounds or environments. Blue *et al.* noted that European carriers have a three times higher OR than the Hispanic population even when they share the same origin of ε4 allele [36]. By genetic screening in specific Arabic populations with high AD incidence, Farrer and colleagues discovered that the elevated AD risk has little connection with ε4 but plausible connections with other genetic or environmental factors [39].

To further understand why genetic polymorphisms local to the *APOE* gene cause ethnic differences, we might first turn to genome-wide association sequencing and phylogenetic research regarding the detection of AD risk factors. To date, the polymorphisms found most relevant to *APOE* and AD are in the sequences of Translocase of the Outer Mitochondrial Membrane 40 (TOMM40) and apolipoprotein C1 (APOC1), two flanking genes on each side of the *APOE* region. In 1998, Lai and colleagues finished mapping SNPs around *APOE* and established a 4-Mb high-density sequence containing 121 SNPs [40]. Later, their team tested these SNPs for their relevance to AD and identified 2 SNPs in the TOMM40 gene showing a strong association with both ε4 allele and AD risk [41]. Roses *et al.* reported one polymorphism, rs10524523, located in intron 6 of TOMM40, defined by the length of its polyT tract, to be closely associated with the age at AD onset [42]. After that, surging amounts of evidence showing the interaction between TOMM40 and AD have been published, suggesting that the TOMM40/*APOE* alleles are better predictors of disease onset than *APOE* alone [43]. Interestingly, the rs10524523 polymorphism is significantly distinct between different ethnicities, which might explain the inconsistent effect of *APOE* polymorphism on ethnicity [43]. Specifically, about half of the *APOE* ε4 alleles of African Americans are linked with the S allele of TOMM40, which is associated with a lower risk or reduced onset of AD than its counterpart, the L allele. In contrast, only 2% of the *APOE* ε4 alleles of Caucasians are linked with the S allele. However, whether the action of TOMM40 polymorphism depends on APOE remains obscure. Although Caselli *et al.* reported that TOMM40 influences the decline in cognitive performance in non-AD subjects in an *APOE*-independent manner [44], more evidence is required to verify the interaction between *APOE* and TOMM40. These two genes are in linkage disequilibrium. Zhou *et al.* reported that variation in APOC1 confers an ε4-independent risk of AD, and the distribution of the APOC1 polymorphism, not surprisingly, varies significantly in different ethnicities [45]. To sum up, polymorphisms in TOMM40 and APOC1 may explain ethnicity-related AD risks, but the underlying mechanisms need further studies.

## Gender and APOE

In medical studies, gender represents the identity defined by the biological distinction between male and female, which results from differences in expression of gender-related genes, gonadal development, and hormone levels [46].

Tremendous efforts have been made to establish the correlation between gender and AD risk. A large population-based study in the USA reported that 2/3 of all AD patients are female, and the longer average life span of women is the most probable cause. In respect of age-stratified AD risk, the specific impact of gender remains debatable. In Europe, most researchers have reported that women suffer a higher incidence of AD, and this phenomenon is more evident in the oldest group (>75 years) [47–49]. Research conducted in Asian countries such as Japan [37] and China [38] found consistent result, while in the USA, most studies on this topic, including the MoVIES Project, the Framingham study, and the Baltimore Longitudinal Study, failed to reach the same conclusion [50–52].

Since the connection of the *APOE* gene and AD was established, researchers have focused on the gender-dependent effect on APOE function. Payami and colleagues first reported that the *APOE* ε4 allele, especially in heterozygous carriers, confers more AD risk on female carriers than males [53]. After that, the meta-analysis by Farrer *et al.*, collecting data from >5,000 AD patients, concluded that female ε4 carriers face a larger increase of AD risk than their male counterparts, as illustrated by the age-stratified OR curve [8]. Subsequent research confirmed this conclusion, and it has become clearer that male carriers with one copy of ε4 have the same AD risk as non-carriers [54, 55]. Besides cross-sectional studies, a longitudinal study by Altmann *el al*., which focused on the speed at which healthy people convert to cognitive impairment during aging, also demonstrated a stronger effect of *APOE* ε4 on women [56]. A recent study analyzing the chromatin accessibility landscape in 19 postmortem late-onset AD brains in comparison with 21 control brains reported that *APOE* loci have more pronounced differences in females than in males [57]. While all the donors in this study were homozygous for *APOE* ε3, it will be interesting to find out whether these gender-dependent differences in the chromatin accessibility landscape have any APOE isoform-specific characteristics.

The gender-dependent effect of *APOE* on AD-risk is evident, but the mechanism behind it remains vague. Fortunately, both clinical and animal studies have provided clues for a possible explanation. It has gradually become clear that the variation of cerebrospinal fluid (CSF) tau levels in patients according to *APOE* gene diversity also occur in a gender-dependent manner. Damoiseaux and colleagues reported a greater elevation of CSF tau, but not β-amyloid, in female ε4 carriers, which coincided with the conclusion of Altmann *et al.* [56, 58]. Hohman *et al.* summarized the information from several large AD cohorts. They reported a stronger effect of *APOE* ε4 in women to cause increased CSF tau, but they failed to find the same difference compared to pathological findings [59]. Later, the same team examined healthy cohorts a with high Aβ burden and found much earlier tau deposition in women than men. Still, this gender-related effect was found to be independent of *APOE* genotype [60]. Besides interaction with CSF tau, gender may influence AD pathogenesis by an estrogenic effect. Specifically, estrogen replacement treatment has been shown to be beneficial for non-ε4 female carriers while it is detrimental for carriers in terms of cognitive performance and AD risk [61, 62]. Estrogen may interact with *APOE* and AD risk by multiple mechanisms. First, estrogen might directly regulate the expression of both *APOE* and APOE receptors. Stone *et al.* found that estrogen replacement treatment up-regulates the *APOE* mRNA level in brain tissue, and Wang *et al.* suggested that this specific regulation occurring in the brain results from the specific distribution of different estrogen receptors in glia [63, 64]. Second, the neurogenetic effect of estrogen is influenced by *APOE* polymorphism. Estrogen has been reported to promote neurite expansion, which only happens when *APOE* ε2 or ε3, but not ε4, is present [65]. Third, estrogen might alleviate the inflammatory response, as NO and cytokine production by immune-activated microglia, and the *APOE* ε4 genotype is reported to inhibit this anti-inflammatory effect [66]. In addition to the tau- and estrogen-related mechanisms discussed above, Ca^2+^ hyperactivity and the gut microbiome have also been reported to be affected by gender-*APOE* association in animal models [67, 68].

## Aging and APOE

Aging is one of the most established and crucial risk factors for AD. Epidemiological studies have found that AD risk increases with age, even in the oldest group (>80 years). The incidence of AD per year gradually grows from 0.6% in people aged 65 to 69 years, to 3.3% in persons aged 80 to 84 years, and even higher in persons aged 85 years and older [69].

*APOE* ε4 acts synergistically with the process of aging, resulting in a distinctive pattern of AD. First, *APOE* ε4 leads to a more severe phenotype of cognitive decline. A pattern of more cognitive decline, mimicking the process of AD, has been found in clinically normal *APOE* ε4 carriers [70]. This cognitive decline was later reported to be strengthened by aging [71]. Second, *APOE* ε4 is associated with accelerated augmentation of AD incidence with age. Qian *et al.* integrated four large cohorts, and their model showed the hazard ratio of AD per year is positively related to the ε4 dose (1.08–1.16, 1.51–2.23, and 2.63–3.57 for 0, 1, and 2 copies) [72]. Third, *APOE* ε4 may have an altered impact on different age groups. A longitudinal study by Bonham manifested a bell-curve association of ε4-related AD risk and age. The strongest effect of ε4 was found in the 70–80 years group, with a peak hazard ratio of 1.8 [73]. However, the difference of ε4-related AD risk across groups failed to reach significance.

*APOE* ε4-related age-dependent AD risk may be partially explained by accelerated deposition of Aβ. Morris *et al.* examined CSF biomarkers and cerebral Aβ imaging in healthy subjects grouped by *APOE* genotype and demonstrated that ε4 carriers show a heavier burden of Aβ42 deposition [11]. Notably, in the 45–49 years age group, the Aβ imaging showed positive findings only in ε4 carriers (10.7% *vs* 0%), indicating that ε4 enhances preclinical AD pathogenesis in adults. Besides, the cortical binding potential of Aβ markers rises with aging in association with the ε4 dose (0.020, 0.013, and 0.003 per year in ε4 homozygotes, ε4 heterozygotes, and non-carriers, respectively), suggesting that ε4 significantly aggravates the progress of AD pathology. Similar effects on other AD pathologies have also been reported in AD patients carrying the ε4 allele [74]. Several studies have reported that a similar pattern of tau deposition is seen in ε4 carriers [75, 76], but this has been challenged by other studies. Proteinopathy of tau and Aβ is widely recognized to act in an age-dependent manner [77]; APOE seems to influence aging-related AD by regulating tau and Aβ metabolism.

## Diet and APOE

Since no current medication can stop or reverse the progress of AD, an increasing number of studies (mainly cross-sectional) have been carried out to uncover the exact role of diet in modulating the course of the disease. Diverse nutrients such as vitamins, antioxidants, and lipids, generally recognized as necessities in brain development and regeneration, came first when searching for AD modifiers. Researchers found that specific types of nutrient impacted the risk of cognitive decline and AD risk. Vitamin B, especially folate and niacin, was reported to be protective against cognitive decline in two observational studies on young adults and older people [78, 79]. Randomized clinical trials testing folic acid supplementation in the elderly revealed the positive effect of maintaining cognitive ability [80]. Vitamin D deficiency, defined as serum vitamin D <10 ng/mL, was shown to be hazardous for AD according to several cohort studies [81], and Zhao *et al.* conducted a prospective cohort study that verified that high vitamin D supplementation is protective against dementia [82]. Omega-3 fatty acids from seafood is another component found to be beneficial by inhibiting cognitive decline. Zhang *et al.* summarized 21 cohorts to conclude that a diet with a higher intake of fish, omega-3 fatty acids, or docosahexaenoic acid (DHA, the major component of dietary omega-3 fatty acids) leads to a lower risk of AD [83], and clinical trials supported the mentally protective effect of DHA in DHA-deficient people [84]. Besides single nutrients, dietary patterns have also been frequently tested for their possible effect on cognitive function. The Mediterranean diet, the Dietary Approaches to Stop Hypertension (DASH) diet, and the Mediterranean-DASH Intervention for Neurodegenerative Delay (MIND) diet have been the major focus of research. Besides fruits, vegetables, and whole-grains, the Mediterranean diet features the consumption of olive oil, plant protein, and seafood, and the DASH diet emphasizes a reduction of saturated and trans lipids, sodium, and sugar intake; whereas the MIND diet is a combination of the former two diets [85]. Numerous cross-sectional studies and several clinical trials have reported that adopting the Mediterranean diet reduces the risk of both cognitive decline and AD [86–88]. Studies focusing on the DASH diet reported that lower sodium intake is associated with better executive functions. Further studies have reported that MIND has a better protective effect against AD than the Mediterranean or DASH diet [89, 90]. Moreover, the ketogenic diet or supplementation with the ketogenic medium are associated with cognitive improvement and a lower risk of AD, with a deceleration of tau and Aβ accumulation in the brain [91, 92].

The benefits gained from diets against AD seem to partially depend on *APOE* polymorphisms. Deficiency of vitamin B12 and vitamin D are both associated with weaker cognitive function based on observational studies, and this is more evident in *APOE* ε4 carriers [93, 94]. Notably, *APOE* ε4 is associated with a lower risk of vitamin D deficiency [95]. The majority of studies examining omega-3 fatty acid supplements reported that its benefits are restricted to only ε4 carriers. In a large longitudinal study of the elderly population conducted by Ondine van de Rest *et al*. [96], weekly seafood consumption with the optimal amount of omega-3 fatty acid intake from food was found to enhance global and several cognitive domains of cognitive function in ε4 carriers. Cross-sectional analyses of deceased subjects reported that weekly seafood consumption was associated with fewer pathological AD findings by autopsy only in ε4 carriers [97]. A randomized clinical trial in younger groups demonstrated that 6 months of DHA supplementation conferred better cognitive performance [98]. Carbohydrate intake could be another dietary factor involved in *APOE*-dependent AD risk. Gendreau *et al.* reported that the glycemic load in the afternoon (mostly representing afternoon snacks) had a synergic effect with ε4 to elevate AD risk [99]. In another recent report, both Mediterranean and MIND diet patterns are more beneficial to ε4 carriers, as was found by Debora *et al.* when examining the association between MIND diet score and cognitive assessment in the Framingham Heart Study [100].

The interaction of AD and diet might function in different manners. *APOE* ε4 has been reported to elevate the serum level of vitamin D [93], indicating a putative protective effect. We postulate that *APOE* ε4 regulates vitamin D transport, conferring resistance to vitamin D deficiency. Therefore, low serum vitamin D in *APOE* ε4 carriers might manifest a more severe undernutrition condition. For saturated fatty acids, Hanson *et al.* reported that the CSF levels of lipids deplete Aβ, which is hazardous for AD pathogenesis, and that this is closely associated with the *APOE* genotype and excessive intake of dietary saturated fatty acids [101]. They proposed that collaboration of the *APOE* ε4 allele and dietary saturated fatty acids leads to less lipidation of CSF Aβ, which results in less Aβ binding to APOE and more deposition of toxic Aβ [101]. For unsaturated fatty acids, omega-3 fatty acids, Yassine and colleagues proposed that ε4 interferes with DHA metabolism, having a neurotoxic effect at an early stage of neurodegeneration [102]. Yassine et al. deduced that: (1) DHA is catabolized faster in ε4 carriers [103], possibly because very low-density lipoprotein is catabolized faster than high-density lipoprotein in the liver, and preferential binding with the very low-density lipoprotein of ε4 thus facilitates lipid transport and catabolism, including DHA consumption; (2) ε4 damages the blood-brain barrier integrity, which inhibits the cerebral uptake of DHA; and (3) ε4 is associated with less lipidation and decelerates the transfer of lipids in the central nervous system. The above led to the conclusion that *APOE* ε4 lowers CSF DHA, playing a crucial role in AD pathogenesis. In addition, DHA is widely known for its anti-inflammatory effect [104] and acts by mediating activated microglia [105]. Bos *et al.* demonstrated that supplementation with DHA through upregulation of peroxisome proliferator-activated receptor-gamma (PPAR-γ), mitigates inflammation in ε4 carriers [106]. For carbohydrates, Zhao *et al.* reported that APOE ε4 in mice impairs the insulin pathway by trapping the insulin receptors in endosomes [107], and hyperglycemia, in turn, facilitates the glycation of APOE and exacerbates AD pathogenesis [108], so that carbohydrate intake elevates AD risk synergistically with APOE ε4.

## Geographical Location and APOE

A limited number of studies indicate that geographical factors modify the pathogenesis or progress of AD. Most studies have reported a positive correlation of residential altitude with the severity of cognitive impairment. In the comparison of a population living at low altitude (500 m), Bolivians living at high altitude (3,700 m) have a slower processing speed and reduced attention, independent of age and ancestry [109]. Hota *et al.* reported that after living at high altitude for one year, acclimatized lowlanders are more susceptible to cognitive decline [110]. Conversely, Thielke *et al.* reported that, in California counties, the mortality rate attributed to AD is inversely associated with the altitude of residence, which fits their theory that long terms of hypoxia might slow the progress of AD [111]. Russ and colleagues explored dementia standardized mortality ratios in Italy and New Zealand and concluded that living at higher latitudes is associated with higher mortality of dementia [112].

Since few studies have concentrated on the interaction between geographic location, *APOE*, and AD, the distribution of *APOE* polymorphisms might help us to deduce how the *APOE* effect is modified by altitude and latitude. Epidemiological data suggest that geographical factors distinctly shape the distribution of *APOE* polymorphisms. In Europe and Asia, the *APOE* ε4 allele frequency is positively correlated with latitude [113, 114]: the lowest value is <10% in the Mediterranean area and South China, and gradually ascends to 25% in northern areas. This gradient suggests that a low latitude might enhance the pathogenic effect of ε4.

It is hard to explicitly determine how altitude or latitude factors affect *APOE*-related AD risk due to the many cofounders such as ethnicity, diet, and economy. However, the geographical distribution of *APOE* may shed light on the mechanism. Vitamin D production by ultraviolet light and temperature account for the major biological effect of latitude. As noted above, the ε4 allele is associated with a higher level of serum vitamin D [115], which may explain why northern populations that receive less UV light exhibit a higher frequency of *APOE* ε4. Eisenberg proposed that temperature may also contribute to the geographic distribution of *APOE* in that people in tropical areas display faster lipid depletion, thus favoring *APOE* ε3 [116]. Although little evidence supports the interaction of *APOE* and altitude, considering that hypoxia-induced cognitive impairment is regulated by the *APOE* genotype [117], *APOE* ε4 may be less frequent in highland populations.

## Conclusions

In this review, we summarized the major confounding factors that might influence the *APOE* genotype-associated AD risk and discussed plausible mechanisms behind these factor-factor interactions. Ethnicity, gender, and age, as observational factors, clearly alter the *APOE*-dependent risk, mainly through variation in local ancestry, hormones, and aging-related proteinopathy, respectively. Diet and geographic location, as interventional factors, are complicated due to their interaction with other confounding factors. However, clinical trials provide evidence verifying that certain subfactors, such as vitamin D, DHA, latitude, and altitude, can influence ε4-related AD risk to some extent.

Since the last several decades have seen repetitive failures to develop Aβ- or tau-targeted therapies for AD, strategies besides decreasing fibril aggregation are gaining popularity, including APOE-targeted therapies. Based on several putative roles that APOE plays in AD pathology, current research mainly focuses on the following strategies: increasing APOE levels and its lipidation [118], blocking APOE and Aβ interaction [119], and using APOE mimetics [120]. We hope the factors discussed in this review may serve to better evaluate APOE-targeted therapies or the grouping of subjects. On the other hand, APOE genotype has been applied in almost all AD risk-prediction models, and researchers are still searching for a better model to elaborate the effect of APOE [121], where stratification by the confounding factors discussed in our review should be the first consideration.

Limitations in this review should be noted. First, considering the wide range of potential factors involved in this topic, certain factors or their corresponding supportive evidence could be missed. Second, studies brought into our review are mostly cross-sectional, with extensively varied study designs and subject conditions, which may compromise our conclusion. Thirdly, as mentioned above, numerous APOE-modifying factors could interact with each other, making the epidemiological evidence less convincing, since the inclusion of all related factors seems impossible in clinical studies. Further clinical trials and meta-analyses are needed for better stratification and regression of numerous factors. Moreover, a surging number of studies concerning APOE and AD is in progress or in the planning stage, and when their results come out, we could have a more comprehensive understanding of this topic.

To sum up, several factors act as a modifier of ε4-related risk, and they deserve more attention for further studies focusing on APOE, from both the investigative and clinical aspects. Since a growing number of therapies targeting APOE are being developed and tested clinically [122], those APOE-modifying factors should serve as new targets for treatment or reference for population stratification.
